# Supplementary material for: Melanoma Cells Inhibit iNKT Cell Functions via PGE2 and IDO1
Source: Cancers (Basel). 2023 Jul 5;15(13):3498. doi: 10.3390/cancers15133498 (PMC10340314; doi:10.3390/cancers15133498)

Fig. 4D

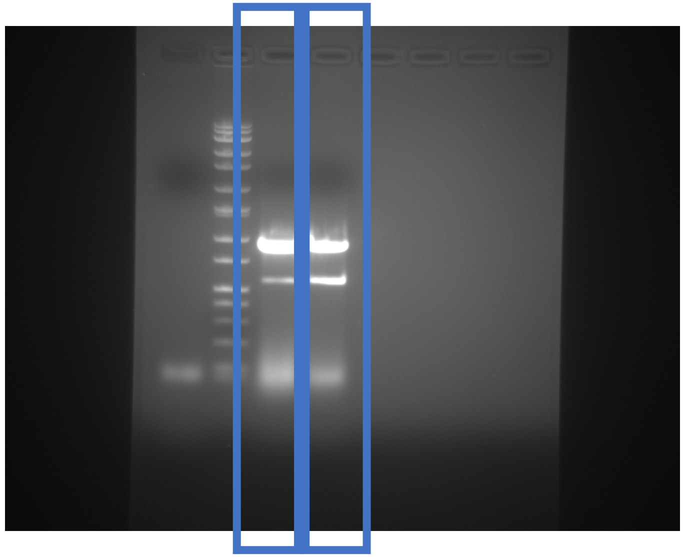

Fig. 4A

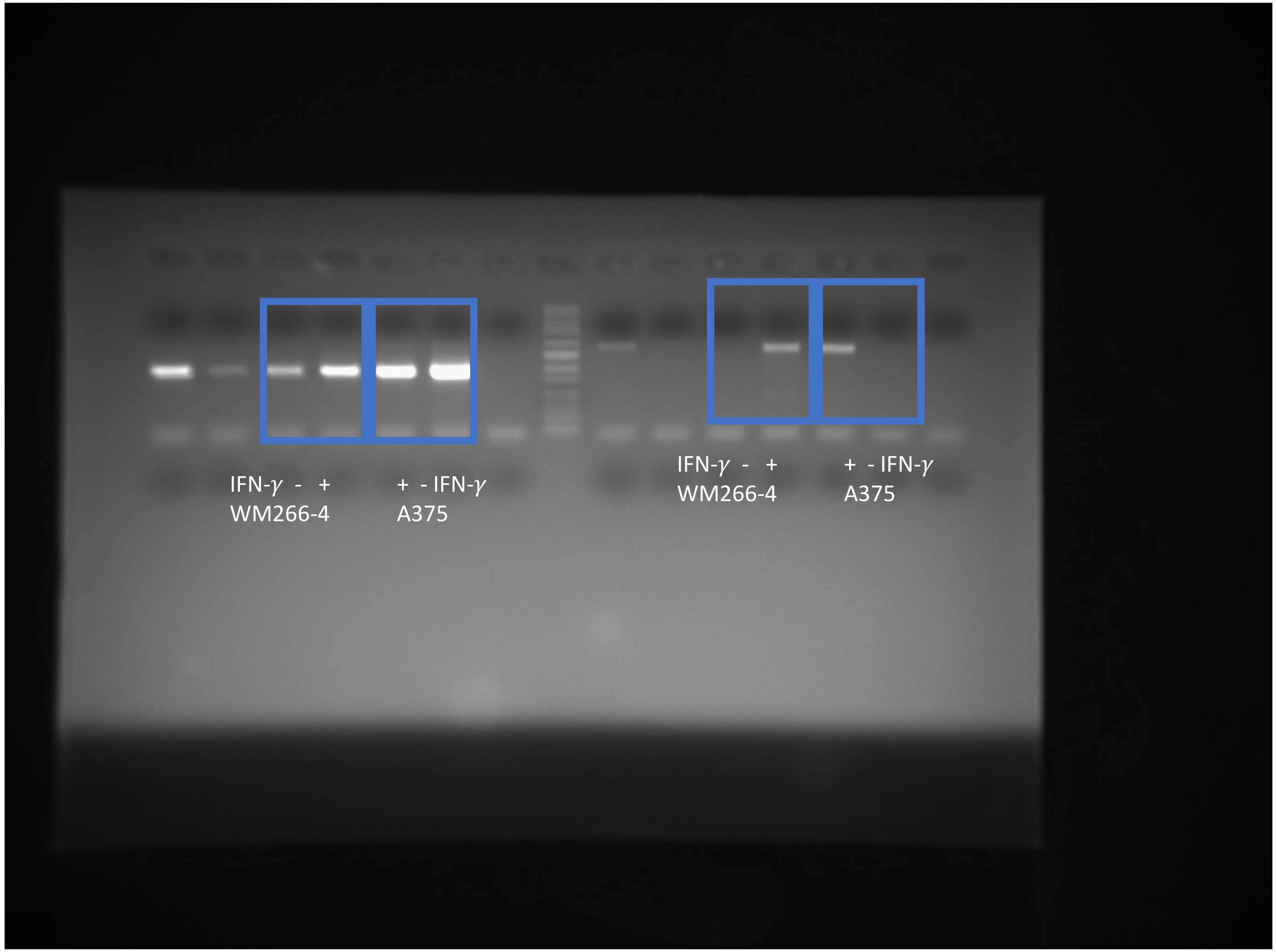

Fig 4C

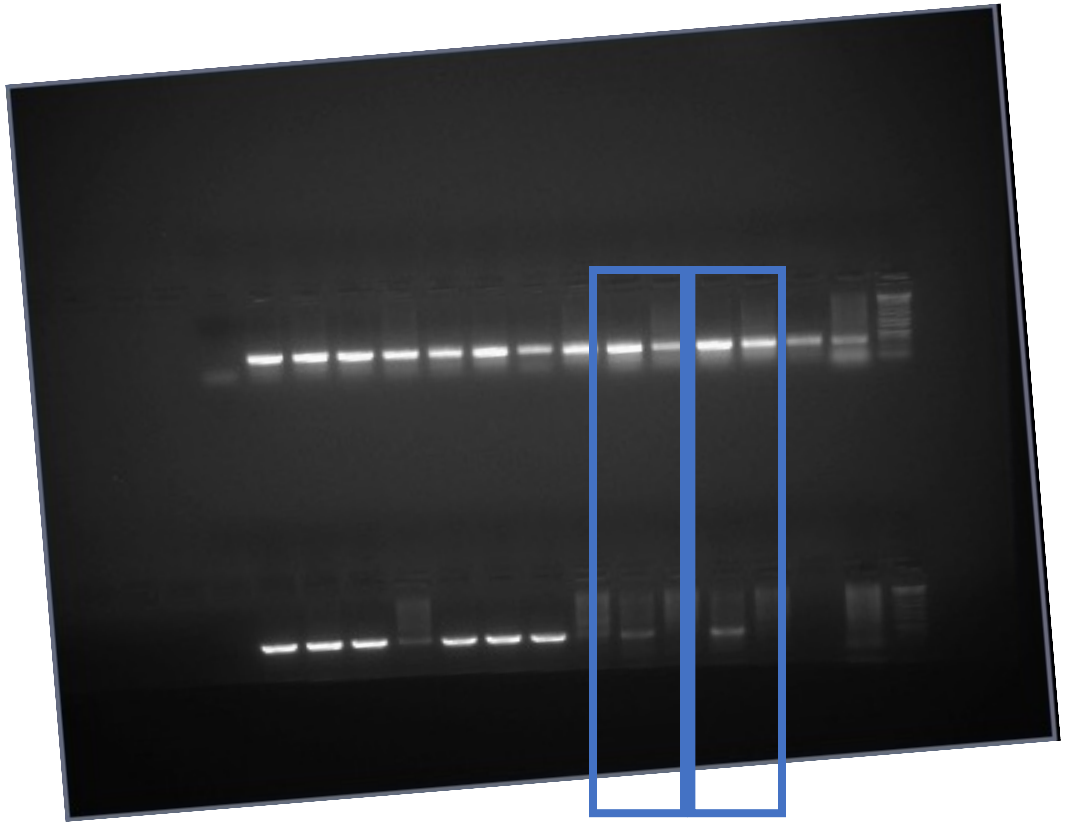

WM266-4 A375
Anti human IFN-γ + - + - Anti human IFN-γ

Fig 4B

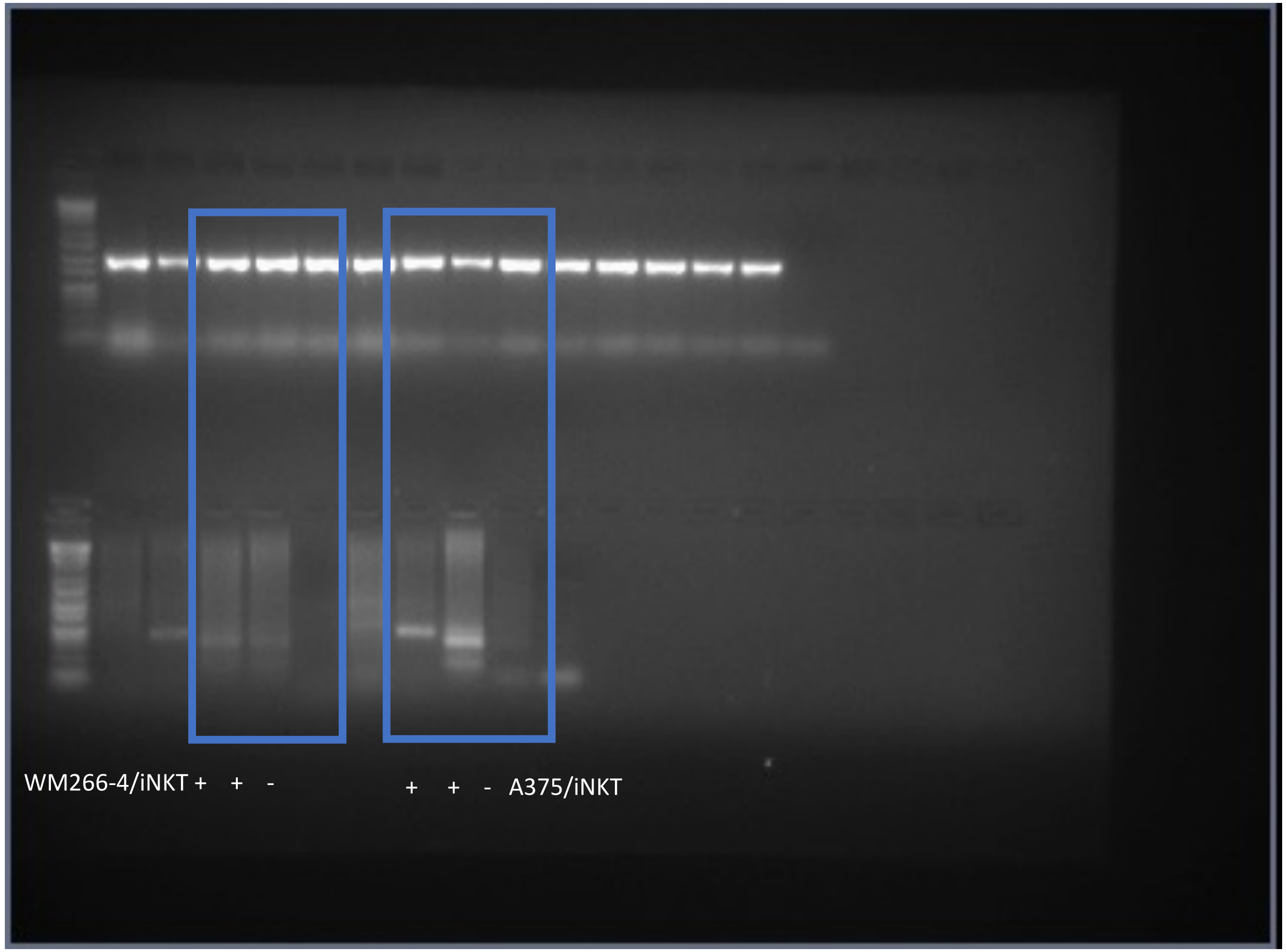

Supplement: Supplementary file 1 [file cancers-15-03498-s001.zip › cancers-2315755-File S1.zip!/cancers-2315755-File S1.pdf]
