# Supplementary material for: Melanoma Cells Inhibit iNKT Cell Functions via PGE2 and IDO1
Source: Cancers (Basel). 2023 Jul 5;15(13):3498. doi: 10.3390/cancers15133498 (PMC10340314; doi:10.3390/cancers15133498)
Supplement: Supplementary file 1 [file cancers-15-03498-s001.zip › cancers-2315755-supplementary.zip!/cancers-2315755-supplementary.pdf]

Supplementary information

Melanoma cells inhibit iNKT cell functions via PGE2 and IDO1.

Authors and affiliations

Enza Torre[1], Giulia Pinton[1] and Silvia Fallarini[1*]

[1] Department of Pharmaceutical Sciences, University of Piemonte Orientale, Novara 28100, Italy

Table S1. PCR primers and protocols used in this study

	Primers	Tm	Product length
IDO	Forward 5′-ACTCCATTGACATCATCTGTGG-3′<br>Reverse 5′-CTCACCAGCAGAATCCAGGAG-3′	60	406
COX-2	Forward: 5′-CGAGGTGTATGTATGAGTGTG-3′<br>Reverse: 5′-TCTAGCCAGAGTTTCACCGTA-3′	60	582
GAPDH	Forward 5′-GGTCGGAGTCAACAACGGATTTGG-3′<br>Reverse 5′-ACCACCCTGTTGCTGTAGCCA-3′	60	1000

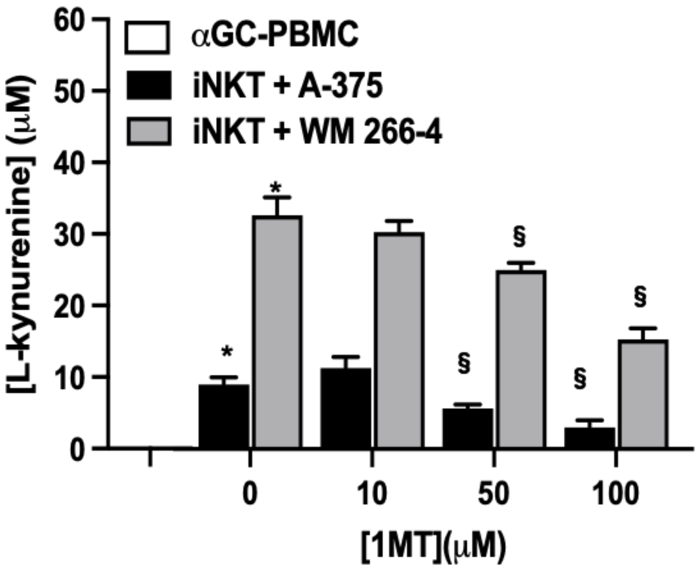

Figure S1 1-MT reduced the kynurenine level produced by melanoma cancer cell lines. Kynurenine concentration in supernatants from αGC-stimulated PBMC cultured without/with melanoma cell lines in presence/absence of increasing concentrations (0-100 μM) of 1-MT. Results are represented as mean ± SEM of at least 6 experiments conducted with PBMC or iNKT cells isolated from different healthy volunteers. * p≤0.05 versus αGC-stimulated PBMC or iNKT cells cultured in absences of melanoma cell lines; § p≤0.05 versus inhibitors untreated co-cultures.

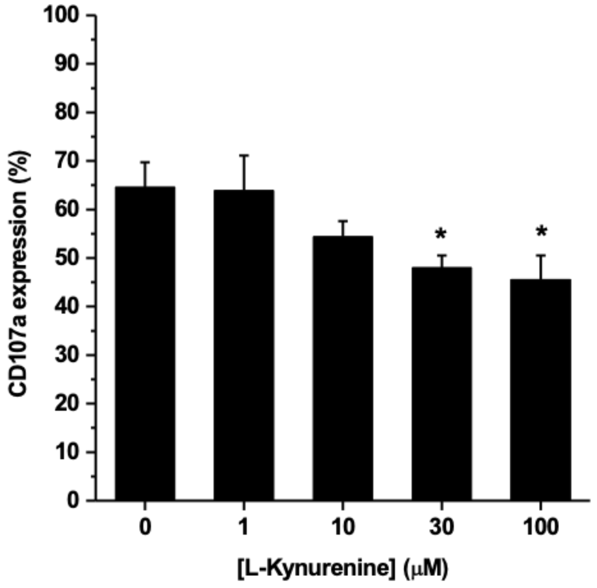

Figure S2. L-kynurenine inhibit CD107a expression on iNKT cells. iNKT cells cultured with increasing concentration of kynurenine (0-100 μM) were analyzed for CD107a expression in redirected degranulation assay against αGC-loaded THP-1. Results are represented as mean ± SEM of at least 6 experiments conducted with iNKT cells isolated from different healthy volunteers * p≤0.05 versus iNKT cell kynurenine untreated-cells

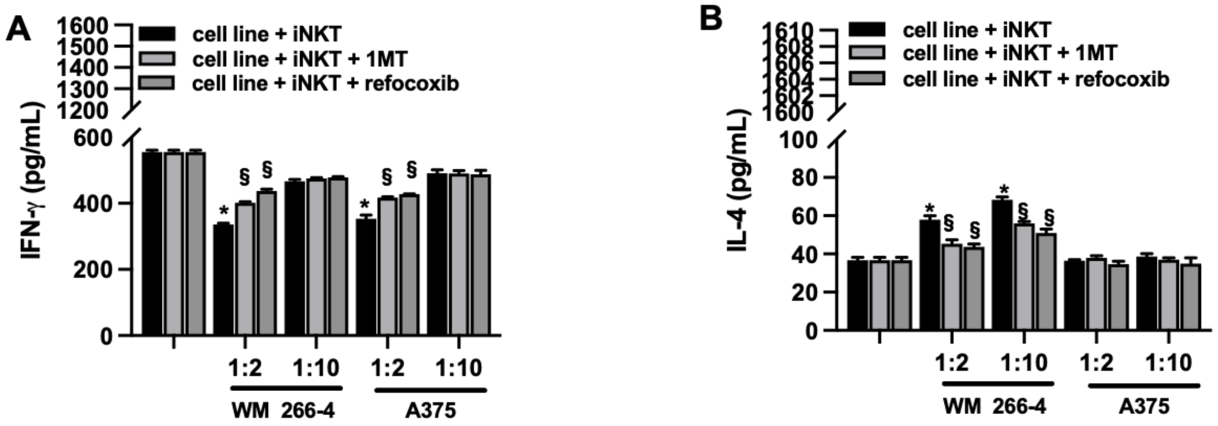

Figure S3 1MT and refocoxib treatment restore the cytokine levels affected by melanoma cell lines. IFN-γ and IL-4 levels were measured by ELISA on supernatants from iNKT cells co-cultured (4 days) without/with melanoma cell lines (different E:T ratio) in presences of increasing concentrations (0-100 μM) of IDO1 or COX-2 inhibitors (1-MT and rofecoxib, respectively) and stimulated (48h) with unloaded THP-1. Results are represented as mean ± SEM of at least 6 experiments conducted with PBMC or iNKT cells isolated from different healthy volunteers. *p≤0.05 versus iNKT cultured in absences of melanoma cell lines. § p≤0.05 versus inhibitors untreated co-cultures.
